# Experiences, perceptions and ethical considerations of the malaria infection study in Thailand

**DOI:** 10.1186/s12910-024-01160-7

**Published:** 2025-01-28

**Authors:** Bhensri Naemiratch, Natinee Kulpijit, Supanat Ruangkajorn, Nicholas P. J. Day, Jetsumon Prachumsri, Phaik Yeong Cheah

**Affiliations:** 1 Mahidol Oxford Tropical Medicine Research Unit, Faculty of Tropical Medicine, Mahidol University, 420/6 Rajvithi Road, Thunphayathai, Bangkok, 10400 Thailand; 2 Centre for Tropical Medicine & Global Health, Nuffield Department of Medicine, University of Oxford, Oxford, UK; 3 Mahidol Vivax Research Unit, Faculty of Tropical Medicine, Mahidol University, Bangkok, Thailand

**Keywords:** Controlled human infection model (CHIM), Malaria, Thailand, Social Science, Bioethics

## Abstract

**Background:**

Thailand has made significant progress in malaria control efforts in the past decade, with a decline in the number of reported cases. However, due to cross-border movements over the past 5 years, reported malaria cases in Thailand have risen. The Malaria Infection Study in Thailand (MIST) involves deliberate infection of healthy volunteers with *Plasmodium vivax* malaria parasites, and the assessment of the efficacy of potential vaccine and drug candidates in order to understand acquired protection against malaria parasites.

**Methods:**

This paper drew from ethics and social science qualitative study called MIST-ETHICS embedded within the MIST studies. MIST-ETHICS aimed to describe and understand the experiences, perceptions and ethical considerations of the MIST studies. Data were obtained from semi-structured interviews and a focus group discussion. A total of 46 participants participated in MIST-ETHICS .

**Results:**

Three major themes emerged: experiences and perceptions of MIST, reasons for joining MIST, and ethical considerations. We found that although compensation was a motivation for participation, this was secondary to it being beneficial to self (health checks; link to health networks; building merit) and others (medical research contribution; altruism). Participants expressed varied opinions regarding the requirement of a university degree as one of the inclusion criteria for MIST.

**Conclusions:**

Our study revealed widespread concerns about long-term health effects and safety. Ethical considerations, including obtaining valid informed consent and ensuring participant inclusivitiy, were deem essential. Despite some debate regarding eligibility criteria, most participants agreed that the informed consent process was robust, accompanied by a strong sense of responsibility to contribute to the greater good. We emphasize the importance of continuously gathering participants’ feedback for quality control, such as improving information materials to clarify the purpose of initial phases, their contributing to later phases, and the rationale behind each selection criterion.

**Trial registration:**

This manuscript is part of the clinical trials registered under ClinicalTrials.gov IDs NCT04083508 (MIST1) registered on 5 Sep 2019 and NCT05071079 (MIST2) registered on 28 July 2021. However, the manuscript pertains to a qualitative study that does not require trial registration.

**Supplementary Information:**

The online version contains supplementary material available at 10.1186/s12910-024-01160-7.

## Background

*Plasmodium vivax* (*P. vivax)* stands among the five Plasmodium species responsible for causing malaria in humans. Vivax malaria leads to some mortality, especially in children, and results in substantial morbidity from relapses, caused by dormant hypnozoites in the liver. Relapses may occur weeks to years after the primary infection. Unfortunately, this disease burden has been consistently underestimated [1]. It is the most widely distributed malaria species and contributes significantly to malaria in South America and Asia. Almost 46% of all cases in South East Asia in 2022 were due to *P. vivax* [40].

In Thailand, *P. vivax* is responsible for more than 80% of malaria cases annually [9]. Thailand has made significant progress in malaria control efforts in the past decade, with a decline in the number of reported cases by more than 55% in 2022 compared with 2015 [40]. However, due to cross-border movements from Myanmar to Thailand since 2021, reported malaria cases in Thailand have risen from 2,426 cases in 2021 to 6,263 cases in 2022. Reported imported cases in Thailand also significantly increased over the same period [40]. Most of these additional cases were diagnosed and treated in the area of Thailand that borders Myanmar, where displaced populations from Myanmar can more easily access health care services. This situation has led to an increase in the required resources for malaria diagnosis, treatment and prevention in Thailand.

Controlled Human Infection Models (CHIMs), also known as controlled human infection studies (CHIS), microbial challenge studies or human challenge studies, involve the careful and intentional infection of human volunteers with micro-organisms under highly controlled conditions [29, 39]. CHIM studies have been undertaken in high-income countries (HICs) for decades, even though most of the diseases pathogens and burden occur in low- and middle-income countries (LMICs) [6, 14]. To date, most human infection studies have been performed in non-endemic high income settings such as the United States, the United Kingdom and Australia, even though for many pathogens most of the burden of infectious diseases occurs in LMICs. These studies have contributed to the understanding, treatment and prevention of a number of diseases including malaria, influenza, cholera, typhoid and hepatitis. CHIMs in Thailand are relatively new. A study on *Shigella sonnei* was one of the first human challenge studies conducted in Thailand [3].

CHIMs for malaria have been suggested, as these allow for the assessment of the efficacy of potential vaccine and drug candidates, and greater understanding of the innate and acquired protection against malaria parasites [18]. The Malaria Infection Study in Thailand (MIST) is being conducted to accelerate the development of vaccines and drugs for *P. vivax* malaria (www.mist.in.th). MIST comprises a series of clinical studies that use CHIM as a platform to test potential vaccines, drugs and other interventions. In brief, MIST involves of three main stages. The first stage (MIST 1) involved infection of volunteers through infected mosquito bite, followed by collection of infected blood for later stages that involved blood stage malaria infected blood human challenge studies. MIST 1 involved laboratory-bred mosquitoes infected with *P. vivax* through membrane feeding on participant blood directly biting and infecting healthy volunteers. After the parasite had multiplied to sufficient levels in the bloodstream, the volunteer’s blood was drawn and banked for future phases of the MIST studies. Immediately following blood drawing, participants were treated with antimalarial drugs until they had fully recovered and two follow-up blood tests demonstrated no active *P. vivax* in the bloodstream. This stage is needed as, unlike *Plasmodium. falciparum*, *P. vivax* cannot be grown in vitro. The second stage (MIST 2) aimed to determine the most suitable inoculum ‘dose’ for a human blood stage challenge in later CHIM trials of blood stage vaccine candidates. For MIST 2, the frozen infected blood controlled from the volunteers who participated in MIST 1 was thawed and diluted into four different concentrations to enable the assessment of safety and feasibility of each concentration for future phases of MIST involving blood stage (as opposed to sporozoite from mosquito bite) challenge. Different concentrations of infected blood were then injected into four healthy volunteers for each of the four dilutions. When participants developed malaria symptoms, they were treated with antimalarial drugs immediately. MIST 2 was conducted in two batches of four dilutions with two participants per dilution (total of 16 participants). Subsequent MIST studies will test upcoming *P. vivax* vaccine and drug candidates. Currently, MIST 1 and MIST 2 are being conducted. Potential physical risks involve arm pain at blood drawing side, being sick from malaria, side effects of the malaria medication, and relapse of *P.vivax* malaria.

To date, only a handful of studies have explored the question of why healthy volunteers join CHIMs in LMICs. These studies found that the biggest motivation were financial incentives. In addition, having a break from work and routine of everyday life, learning more about science and medicine, and curiosity in infection research were mentioned as motivations [22, 29]. The potential scientific and medical benefits of CHIMs are significant. However, such studies are ethically sensitive, especially in LMICs, where safety procedures need to account for potential pre-existing immunity in endemic countries [6]. Limited understanding of the awareness, attitude and perception on the infected healthy volunteers with parasite and the use of blood samples during trial studies can have negative effects on the conduct of these trials, potentially impacting the success of the of clinical research [10]. Conducting MIST is important for a number of scientific, practical and ethical reasons. The findings in healthy *P. vivax* naïve volunteers in HICs may not be able to be extrapolated for future vaccine deployment in Thailand and other countries in this region. There are clear genetic differences and greater heterogeneity of *P. vivax* immunity in countries where malaria is endemic. MIST studies will enable a better understanding of the biology and transmission of *P. vivax*, and provide an experimental clinical platform for the study of new vaccines and therapeutic. Moreover, as part of the target population for interventions, the volunteer population in Thailand is an ideal demographic for testing the novel vaccines, medications and their distribution.

## Methods

This paper drew from the results from our ethics and social science qualitative study called MIST-ETHICS, embedded within the MIST studies. MIST-ETHICS aimed to describe and understand the experiences, perceptions and ethical considerations of the MIST studies.

### Study setting

The MIST-ETHICS study was implemented alongside the MIST 1 and MIST 2 studies conducted at the Hospital for Tropical Diseases, Faculty of the Tropical Medicine (FTM), Mahidol University, Bangkok, Thailand. The research institutions involved have a longstanding and productive collaboration, including on malaria research, and are trusted and respected in Thailand. FTM, Mahidol University, has good facilities, malaria expert, and ready access to ‘native’ *P. vivax* malaria parasites from a Bangkok laboratory, removing the need to import the parasite from other countries.

### Participant selection, recruitment and profile

**Fig. 1:**
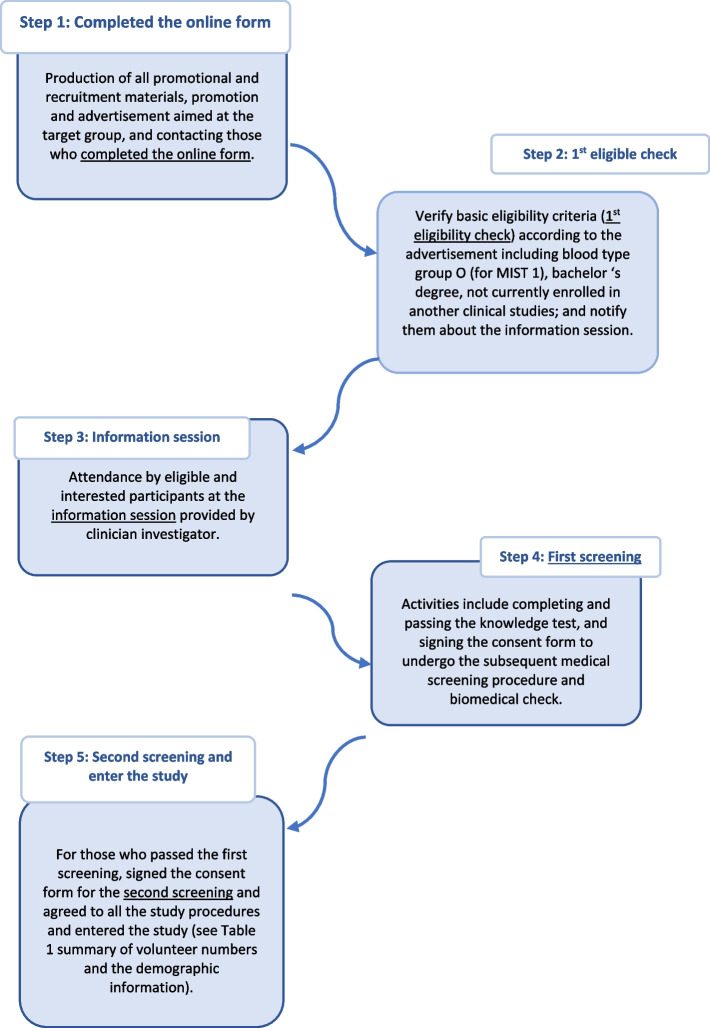
Recruitment process for MIST 1 and MIST 2 participants

The two main groups of MIST-ETHICS participants were those enrolled in the MIST study (Group 1) and non-MIST participants (Group 2). The recruitment process for MIST 1 and 2 participants is demonstrated in Fig. 1 and the summary of people reached at each recruiting step is presented in Table 1.

**Table 1:** Summary of numbers and demographic information of MIST 1 and MIST 2 participants from Step 1 to Step 5

	Step 1: # of people who completed the online form	Step 2: # of people who passed 1^st^ eligibility check	Step 3: # of people who attended infomation sessions	Step 4: # of people who attended first screening	Step 5: # of people who entered the study
**MIST 1 - total**	346	118	94	46	4
Gender (men/women)	121/220 ( no gender identified for 5 participants)	51/67	32/62	17/29	1/3
Number of people who obtained bachelor degree and above	240 (106 did not have bachelor degree)	118	94	46	4 (including 1 with Masters degree)
**MIST 2 - total**	140	86	62	43	16
Gender (men/women)	42/98	29/57	22/40	17/26	4/12
Number of people who obtained bachelor degree and above	120	86	62	43	16 (including 1 studying PhD)

### Group 1

MIST-ETHICS participants were healthy volunteers who had enrolled in MIST 1 and MIST 2. Given the complex nature and perceived elevated risks of the first CHIM malaria study in Thailand, the FTM Ethics Committee recommended that participants should possess at least a Bachelor’s degree to ensure a comprehensive understanding of the study. MIST 1 enrolled 4 participants, all of whom participated in the MIST-ETHICS study. MIST 2 enrolled 16 participants, with 13 of them consenting to be interviewed in the MIST-ETHICS study. Each interview was undertaken at the study site.

### Group 2

Non-MIST participants comprised of four sub-groups. A purposive recruitment sampling frame was applied for this group according to specific characteristics, described below.

#### G.2.1

People who had expressed interest in participating in the MIST studies and attended the clinical screening, but had not met the eligibility criteria. Individuals who were considered ineligible due to conditions such as anaemia, high cholesterol or any blood-related diseases were informed about the MIST-ETHICS study and asked whether they would be interested in participating in an interview. The interviews were performed within two weeks of receiving the test results and took place at the study site.

#### G.2.2

They were family members and friends of participants in Group 1. All enrolled MIST 1 and MIST 2 participants were informed about the researchers’ interest to interview family members or friends, and they were asked to pass a form to those interested. The interviews were conducted by phone after the Group 1 participants had returned home.

#### G.2.3

They were people from the wider community who were interested in participating in focus group discussions on the social, ethical and clinical aspects of controlled human infection studies in general. At the end of the information sessions for MIST 2, which were conducted from February 2022 to October 2022 (see Fig. 1, Step 3), an announcement that a focus group discussion would be held was made. Interested participants expressed their interest to staff, who sent them the meeting link. The group discussion was conducted online.

#### G.2.4

There were key informants (KIs) who were professionals with technical knowledge and understanding of CHIMs and the ethical aspects of the clinical studies, and in decision making positions. They were purposively selected according to their professional interests, and include malaria and infectious disease researchers, media representatives, lawyers, human rights activists, research ethics committee members, and staff involved in the MIST clinical studies. All participants from this group were contacted directly from the authors’ network and interviews were done in-person.

In total 46 participants were recruited in into the MIST-ETHICS study from November 2020 to October 2022. See details in Table 2.

**Table 2:** Details of each group of MIST-ETHICS participants

Group	Description	Number of participants interviewed (total number of interviews)	Number of women (W) / men (M)
Group 1 (G1)	Participants enrolled in MIST 1	4 (total number of interviews: 14)	W: 3 / M: 1
	Participants enrolled in MIST 2	13 (total number of interviews: 35)	W: 9 / M: 4
Group 2.1 (G2.1)	Individuals who were interested to participate but had not met the MIST 1 or 2 eligibility criteria	9 (total number of interviews: 9)	W: 6 / M 3
Group 2.2 (G2.2)	Family members and/or friends of Group 1 participants	4 (total number of interviews: 4)	W: 0 / M: 4
Group 2.3 (G 2.3)	Wider community	10 (1 focus group discussion)	W: 7 / M: 3
Group 2.4 (G2.4)	Key informants	3 ethics committee members (total number of interviews: 4) 1 media person (total number of interviews: 1) 2 MIST project staff (total number of interviews: 2)	W: 3 / M: 3
Total		46	W: 28 / M:18

### Theoretical framework, data collection and analysis

MIST-ETHICS is underpinned by an ethical framework for biomedical research, including social value, scientific validity, fair participation selection, a favourable risk-benefit ratio, independent review, informed consent and respect for participants [13]. Study information was made available to the public in layperson’s language. For this, we sought the help of the Health Research Ethics Interest Group (HREIG), an independent public advisory group based in Bangkok.

Qualitative research methods and a thematic descriptive approach were used in this exploratory study [4, 11, 21]. The use of a qualitative descriptive design was considered the most appropriate approach, as there is not yet a deep theoretical context for this research in Thailand, perceptions and experiences of the participants are highly subjective, the results were to be presented to closely mirror the expressions of the participants [12, 35], and the overall aim was to contribute to change and quality improvements in clinical and public health settings [7]. According to McMillan, et al. [26], emphasis was placed on the reduction of participant objectification by minimizing subjectivity and focusing on the experiences and interests of participants in being involved in the research.

The qualitative data were obtained from semi-structured interviews (Group 1, Groups 2.1, 2.2 and 2.4) and a focus group discussion (Group 2.3). An interview guide was developed for this study only [27]. Informed consent to participate was obtained from all participants before each interview or FGD began. All interviews were conducted (by SR, NK and BN) in Thai language. Group 1 participants were interviewed multiple times, with the majority having three interviews. The first interview took place after hospital admission, but before malaria symptoms appeared. Additional interviews were conducted once or twice after recovery. Some participants were also interviewed during follow-up visits after discharge. The first interview aimed to understand the preparation the participants undertook to take part in the study, motivating factors, the informed consent process, and their understanding and perception of deliberate infections in general and specifically the MIST procedures. The second interview was conducted after they were treated with the anti-malarial drugs and had fully recovered from their malaria symptoms. Participants’ lived experience including illness, treatment and care, perception and understanding of MIST, the concept of deliberate infection, and compensation for participation were explored. The last interview was to explore participants’ reflections about what had happened to them, their concerns about their own health, concerns on whether they could infect other people, their experiences after they had returned home, and whether they have shared their experience participating in research with others.

Non-MIST participants (Group 2) were included in order to develop a comprehensive understanding of the views and perceptions of the broader community, to test validity through the unification of different data sources and to strengthen and support any additional information from all other groups for triangulation [5, 23]. All Group 2 participants except Group 2.3 were interviewed once. Participants who had not met the MIST eligibility criteria (Group 2.1) were asked about reasons for wanting to participate, how they felt about not meeting the selection criteria, and their understanding of MIST and deliberate infections in general. Family members and/or friends of Group 1 participants (Group 2.2) were asked about their role and involvement in decision-making for Group 1 participants, perceptions about risks and benefits, and ethical issues about MIST more broadly. Members of the wider community who were interested in discussion of the social, ethical and clinical aspects of controlled human infection studies (Group 2.3) were invited to participate in a group discussion to explore broader social and ethical aspects of CHIM. Key informants (Group 2.4) were asked specific questions relevant to their areas of expertise. These included perceptions of the concept of deliberate infection and MIST, their understanding of motivations for participation, and ethical guidelines for CHIMs in general.

Detailed data analysis were conducted by the interviewers (BN, NK and SR). This involved reading through all transcripts, making sense of the data, assigning codes, looking for patterns in the meaning of small codes, and grouping them into themes. This initial data coding was performed independently by each core team member (BN, NK and SR). Once each person had completed the coding and identified themes, the themes were discussed with the wider team and refined where necessary.

## Results

Three main themes emerged: 1) experiences and perceptions of MIST; 2) reasons for joining the study; and 3) ethical considerations. The following section gives a description of each theme broken down into sub-themes. We provide illustrative quotes, noting de-identified participant reference groups, reference numbers and gender of participants (‘G’ represents the participant group mentioned above, ‘P’ represents the person’s references number (P1, P2, P3….) and ‘W’ or ‘M’ represents gender.

### Theme 1: experiences and perceptions of MIST

#### Preparing before entry to the study

All Group 1 participants were provided with full and detailed explanations about the MIST trials. After the information session (refer to step 3 in Fig. 1), participant Information Sheet (PIS) was made available on request for all attendees. Additional sources of information including MIST 1 and MIST 2 YouTube videos which explained the study’s procedure, were also mentioned. These resources were provided to help those interested in MIST studies gain a better understanding of what would involve and the potential impact on their health. Most Group 1 participants informed us that they had then undertaken additional informal online research (e.g., through YouTube and various websites) prior to making the decision to join the study. This was to gain a further understanding of what would happen and the likely impacts on their health. They searched, in particular, for more information about malaria and the symptoms and treatment of the disease, and about CHIMs.

“After I listened to the doctor at the information session, I went back to study and found information by myself through YouTube (https://www.youtube.com/@mist578) to assess the risks and whether entering the project was good or not.” (G1, P13, M).

Some participants consulted with their family and/or friends to help or support their decision to join the study.

“I went to consult my brother. He was worried about it and did not want me to join the project. My parents felt the same way. But after receiving information from the doctor via the Zoom platform, everyone was relieved of their worries.” (G1, P5, W).

As one of the selection criterion was to be healthy, most participants reported preparing themselves well before joining the study by eating healthy food, avoiding unhealthy food and drinks, and doing more exercise.

“Normally, I’m already a person who exercises, so I don’t have to make a lot of adjustments. Another extra attempt, as being suggested by a research nurse, is reducing spicy food and not to take supplements.” (G1, P13, M).

#### Understanding the MIST studies

Through a strict consenting process, which all MIST participants required to complete before joining the studies, and with the support of project materials such as PIS and video, all MIST 1 participants demonstrated a good understanding of the procedures and risks. They were able to explain the study in detail and correctly.

“The first day that I joined the study, I thoroughly read the documents that the doctor gave me. The next day we went to the lab and were bitten by mosquitoes to get infected with malaria. The nurse took our blood every day to see how the infection was going. Then, we got sick and donated the infected blood. The doctor gave us medicine to treat malaria immediately. We were discharged after we fully recovered, but had to come back to hospital every day to take drug up to 14 days. We came back for appointments on Days 28, 45, 90 and after 1 year. For the six months after joining the research, I had to stay in Bangkok.” (G1, P1, W).

All MIST 2 participants who were interviewed correctly explained the process and risks of deliberate infection. They demonstrated an understanding that they would likely become ill with malaria following the injection of *P. vivax -*infected blood, and that some of them would be receiving higher concentrations than others.

“The first step is infecting healthy volunteers with malaria, which is in the red blood cells. It is understood that the second phase of the experiment involves dividing the malaria infected blood into four concentrations, injecting into four volunteers, and then check how each person’s body responds.” (G1, P3, W).

Participants also expressed the understanding that those infected with the highest concentration of *P. vivax* infected blood would likely become ill with malaria more quickly than those who received diluted blood. They also understood that MIST 2 was about testing the malaria concentration in the blood for the next study phase.

“There were four tubes of blood. Each one had a different amount of malaria in the blood. One tube is quite red and has more malaria than the others. I can see that from the colour of the blood in the tube.” (G1, P4, W).

“The study is about testing of *P. vivax* concentration levels in the blood for future studies.” (G1, P3, W).

However, 2 of 13 MIST 2 interviewed participants had some misunderstanding about the purpose of MIST 2. They thought that MIST 2 was for drug-testing rather than a study aimed at determining the correct infected blood dilution level for future stages of the MIST programmes. Hence, they mentioned that the medications provided by the doctor after they developed malaria symptoms were effective in treating the disease, and they felt better after taking those medicines. This misunderstanding was clarified at the end of the e\interview, ensuring they fully understood the purpose of MIST 2.

“You can tell the doctor that the medicine that I am taking is working well. This formula can be used to treating malaria.” (G1, P10, M).

#### Safety and risks of participation

The most common concern of enrolled participants and people who were interested in participating in MIST was about the long-term effects that they might have after getting malaria.

“I was a bit worried when I heard that the volunteer will be injected with malaria because I don’t know what the effect on my body will be, or how my body will react to the germ. While I will be in the study, it will be okay because I will be under care from the doctor. My concern is the long-term effects after the project finishes, whether I will still have any malaria remaining in my body. However, I am not very worried.” (G2.3, P5, W).

Some participants expressed fear of the injected blood, and that it could be contaminated in some other way.

“At the time that I was about to be injected with malaria-infected blood, I was not afraid of malaria, but I was worried about where the blood came from. I came to know later that the blood in the tube came from MIST 1. I am still worried whether the blood was clean and safe or not.” (G1, P4, F).

There are concordant views towards safety and risks for both experts (Group 2.4) and family members (G2.2). They expressed that the overall MIST study was safe and well planned for mitigating the risk.

“Malaria is not an emerging disease. It already has effective drugs for its treatment. If this study is about testing a new drug for a new disease that has no treatment and these volunteers are the first experiment group, I would feel very worried.” (G2.2, P2, M).

### Theme 2: reasons for joining the study

The results from both enrolled in MIST and non-enrolled participants suggested that two main and equally important reasons underpinned participation: benefits to self, society and altruism; and financial compensation.

#### Benefits to self, society and altruism

Participants spoke of the importance of gaining the motivation to achieve better health, maintain good health and adopt a healthier lifestyle, including eating healthy food, good sleeping pattern, and a lifestyle of slowing down.

“After joining this project, I felt that I was eating better and that was making me healthier. I’m sure that when I went back to cook at home, the taste of the food I cook will be milder -- not too sweet or salty.” (G1, P8, W).

Any participants interested in participating in the study had to undergo a physical health checkup, which as part of the study procedures is free, to determine eligibility. The free health checkup was mentioned as one of the benefits by those who participated in this study. Though all Thai people can access any public health service under the universal health coverage scheme, knowing a physician directly could have many benefits, especially reducing the waiting time to see the doctor.

Connections with the health system and medical professionals were appreciated and acknowledged as of current and future benefit.

“I am now 22. I think there might be a long-term benefit. When I am 27-28, if I have any health problems, I will contact the medical team again.” (G1, P5, W).

The benefit to self (worthiness) extended to feelings of benefit to others and to society. Many participants articulated their involvement as an important contribution to innovation and humankind, being part of medical history, and their desire to see the successful development of a good vaccine that would make people healthier and save lives.

“I see myself as a living medical textbook. If no-one was sick, how would the doctor understand the treatment? Assuming that no one is sick from [vivax] malaria, how could the doctor know what to do? Reading from text book is only giving knowledge but reading [documenting] the study is a real-life practice. This method of study is the right way, and we are the last book that the doctors need to understand.” (G1, P10, M).

The concept of altruism or selflessness was mentioned by most participants, regardless of religious and belief backgrounds. The majority were Buddhists and expressed their volunteering in MIST as the biggest *tam bun* (merit making) a human can do in this life. A Muslim participant said:

“Here is the thing: you donate blood, you can only give it to one person. Islam loves those who sacrifice for the greater good. My husband said that what I did today was the biggest *bun* (merit) I can do. Islam believes in *zakat*, which means we share things to those in need. It’s a duty, which is taken from our total income. This is out of our duty, so it becomes merit (zakat). I have helped people before, but today I am helping people at a much bigger scale.” (G1, P9, W).

#### Financial compensation

During the recruitment stages for MIST, one of the most frequently asked questions from interested participants was the total amount of money they would receive as compensation for participation. The compensation guideline includes, for example Thai Baht 2,000 for the first screening, Baht 1,500 for the second screening, Baht 2,000 per day for hospital stay, and Baht 1,500 for travel cost to the hospital for each follow up visit. The total compensation amount each participant receives varies according to how many days they stay at the hospital. The range of compensation is between Baht 58,500 to Baht 70,500 for MIST1 and Baht 39,500 to Baht 51,500 for MIST2. Group 1 participants were asked whether they joined the study because of the money. All demonstrated reluctance to answer this question at first, but after probing, they confirmed that the compensation contributed to their decision to join the study. Most clarified that the receipt of financial compensation was not the primary or most important reason, but that it was important to compensate for risk and longer-term health impacts.

“After getting infected, we may have fever and get sick. The compensation had to be commensurate because we put ourselves at risk. I think the amount is good, not too much and not too little, because we stay in the hospital for half a month, which is quite a long time.” (G2.1, P2, M).

As the compensation was equivalent to payment when working, some perceived participation in MIST as similar to being employed, with their role and responsibilities mentioned in the Participant Information Sheet (PIS) that they had signed.

“Any area of work requires resources – people, money, and time. Time is money. The role of being a volunteer and providing information to the study (being examined and data source) is equivalent to the time investment of the volunteer. Therefore, it requires compensation for time and should be appropriate.” (G1, P8, W).

When asked about their understanding of the guided reference for compensation rate, some participants interested in MIST studies referred instead to the insurance compensation rate. In addition to universal health coverage provided by the government to all Thai citizens, many people purchase private health insurance to cover loss of income when off sick for more than 12 h, and almost all participants felt that the amount of compensation received was fair and reasonable.

“You can compare with the insurance rate; it should be around 2,000 to 3,000 [Thai] baht per night if you stay at the hospital. After I heard about the compensation rate from the information session, I did some research. The compensation rate that the project pays is similar to those of insurance companies. It is reasonable.” (G2.1, P5, W).

### Theme 3: ethical considerations

#### Consenting process

All participants thought that the consenting process was carefully conducted. They acknowledged the importance of answering all questions correctly (refer to Fig. 1, Step 4) in order to understand the project, and the need to explain the processes and their involvement before the first screening. With prior perceptions of risk, the test helped ensure that all participants felt that they understood what would be happening to them.

“I can remember the day I came for the first screening. I had to do the test first. I think that was very good, because I got to test how well I understood the project. […] I read the document (PIS) and signed it, and think it (PIS) was sufficient.” (G1, P15, W).

Married participants mentioned discussions with their spouse before consenting to help them come to a decision.

“When I told my wife that our daughter and I were going to a screening for the study, she felt very worried and anxious. She asked me about the project. I explained to her all the steps and procedures of the study. After that she felt relieved and felt that it was okay for both me and my daughter to join.” (G 2.1, P3, M).

#### Disclosure to family and friends

All participants were required to provide the contact details of at least one person who was to be contacted in case of emergency. Many participants who reported themselves as single or not married did not want the study team to contact family members in an emergency, as they had not told them about their participation. To explain their absences, some told their parents that they were away for a few weeks for work purposes or to attend a camp. They mentioned that family and friends who had not undergone the thorough learning process of the risks might not appreciate the participant’s full and comprehensive journey to understand and agree to participate. They did not want to cause any unnecessary worry to family members and friends.

“I didn’t tell anyone at work, as I felt this was my own confidentiality, as well the project’s. I told other people and people at work that I would be traveling to another province for work purposes. Different people have different ideas. They might think the project is too dangerous and worry about me. (G1, P13, M)

Many participants did not disclose their involvement or gave scant details about the study to friends and family. Some mentioned that the study was complex and difficult to explain, and that they did not want to enter into such depth and detail in their conversations, particularly if this could lead to misunderstanding or negative emotions. Therefore, some participant expressed reluctance to have their family or friends interviewed.

“I told my cousins and siblings, but not my mother. I am afraid she might ask a lot and it is hard to explain to her. She would be worried, especially if she knew that I was here to be infected. The elder doesn’t really understand, my siblings are more open. I told my younger relatives, and they asked me if I was not scared. They know that I made the right decision.” (G1, P2, W).

Some participants mentioned that they might inform their family and friends about their involvement following the completion of the study, and their survival and return to full health.

“I will wait until the end of the program to tell my mum when I see her. She would be very worried if I just texted her on a LINE application [a common social media application in Thailand.]” (G1, P8, W).

#### Inclusiveness and fairness

Many points were raised about the eligibility criteria and whether these led to the exclusion of populations who might benefit from participating in the research. The requirement to have a university degree especially led to much debate. There were some disagreements on whether having a university degree was a good predictor for understanding the study. Consider these two quotes:

“I think people who have higher education can understand the project more than those who have lower education. The study is complex. You need to understand the project before making the decision.” (G2.1, P7, W)

“I don’t understand the project deeply even though I am already in the field (nursing) and graduated with a bachelor’s degree. For example, I don’t deeply understand the scientific process of mosquitoes and what will happen next” (G1, P2, W).

Some questioned whether the inclusion of a degree in any discipline as an eligibility criterion was the ‘best way’. Those who had obtained a non-health-related degree might not be able to understand the science and risks as well as those who had undertaken health-related courses.

The ethics committee strongly insisted that at least a bachelor’s degree be included as one of the selection criteria to ensure participants’ comprehension of the study. However, some participants, particularly those who could not meet criteria for MIST, expressed concerns that ondovoduals without a degree (potentially due to lack of opportunity) might still be equally capable of understanding the complexities of the study’s concepts and methods. They pointed out that this was especially through given the clarity of the information materials and the use of layperson-friendly language.

“If you can reduce the qualifications of volunteers to M.6 (equivalent to high school level), it will give more opportunity to more people who are interested in joining the project. (Lack of) qualifications do not reduce the quality of people.” (G2.1, P1, M).

## Discussion

The MIST programme’s aim is to accelerate the development of vaccines and drugs for *P. vivax* malaria. The MIST-ETHICS study provided continuous feedback to the wider MIST project team. Three main themes emerged: experiences and perceptions of MIST, reasons for joining the study and ethical considerations.

The strict eligible criteria for participation in MIST were seen by some participants as being at the expense of inclusivity. From a scientific perspective, inclusiveness is important to ensure that trial participants are appropriate and representative of the target populations for vaccine and drug/therapeutic development [38]. Moreover, inclusiveness protects the ethical imperative principles of equity, diversity and respect for individual [2]. MIST-ETHICS participants appeared to suggest that the exclusion of those who did not meet the strict selection criteria meant exclusion from the benefits of participation (for example, not having the opportunity for financial reward or medical checkups, or to build *tam bun* [merit]). The strict criterion of education level of at least Bachelor’s degree has been controversial among MIST-ETHICS participants. Jamrozik & Selgelid [19] argued that selection criteria of higher-level education raises several ethical concerns: individuals with lower levels of education do not necessary have a limited or low understanding of the study and the informed consent process; and excluding less-educated individuals can be seen as unfair, especially if they are at high risk for the condition be studied.

More women than men participated in the MIST studies. The reasons for this were not explored fully in MIST-ETHICS. Numerous studies suggested that higher female participation in clinical studies [24] is influenced by gendered norms, especially women’s roles in care work, with women primarily responsible for household chores and the health and wellbeing of children, adults requiring significant care, and other family members [25] and any opportunities that women could contribute to income generation including participating in clinical trials UN Women, 2020)[37].

Our findings reflected similar motivations to participate in CHIM studies conducted elsewhere. These studies found that altruism [34], financial compensation [30], scientific development [32] and self-satisfaction [28] were common reasons. Almost all religions encourage people to serve others [33], and this is likely to have influenced Thai volunteers. Volunteering and the virtues of caring for others are inherent to Thai culture and rooted in the beliefs of religious personhood [31].

The question of how fair compensation for research participation is determined is ongoing [16]. Undue inducement and the potential for exploitation need to be balanced with a fair and reasonable amount of financial and other compensation for participation [15, 17]. Because trial participants were compensated, researchers were concerned about perceptions of exploitation, particularly of people from lower socioeconomic circumstances. However, no interviewees mentioned exploitation. Analysis of the perceptions of MIST participants suggests that a reasonable balance was achieved. No participants mentioned compensation as being too little or too much, although this could be because they were hesitant or embarrassed to speak of finances. Additionally, they might have been reluctant to share their true perception about compensation amount because three researchers were present during interview. Although compensation was considered to be important, it appeared secondary to other benefits to self and others. Consistent with results from previous CHIM studies [22, 29], participants mentioned interest in joining MIST to learn more about the medical management of malaria and contemporary infection research, to gain access to health care, and to contribute to science [36]. Consistent with Thailand cultural values and norms (e.g., salaries are not revealed in recruitment advertisements), the advertisements for MIST did not mention the total compensation amount. This was to minimise the chance of exploitation and ensure that those who were desperate for money only did not apply. The amount of financial compensation was only revealed to people who were interested in the study during a phone call with the study staff.

We found that many participants preferred not to inform their families about their participation, or gave scant details. In many cases this was verbalised as not wanting to worry others, possibly a manifestation of the Thai cultural practice of *krengjai*. Wyatt and Promkandorn [41] proposed ‘*krengjai’* as the most difficult Thai cultural concept to translate into other contexts, but likely had a significant influence on the conduct of and interpretation of the results from research in Thailand. They noted its meaning in English as encompassing consideration for others, shyness, deference to losing face, and the maintenance of social order/smoothness [8, 20]. Another reason for not informing relatives could be related to stigma. Being (or being perceived as) in financial difficulty is a stigma in Thailand, and some participants mentioned that they did not want their family to think that they needed the money or that they were poor. Carrying a disease and the risk of giving malaria to others might also have been a perceived stigma. While some reported feeling proud to be part of medical history, this was not of sufficient weight to inform relatives before participating in MIST.

### Limitations

*The strict selection criteria* of Bachelor’s degree, introduced potential selection bias and raised ethical concerns regarding inclusivity and equity. Excluding individuals with lower educational levels may have limited the diversity of perspectives, particularly those from high-risk populations. This reflects a broader challenge in balancing scientific rigor with fairness in participant recruitment. The study did not fully explore *gender dynamics* to understand why more women participated than men. While gendered norms, such as women’s roles in care work and contributions to family income, were acknowledged, they warrant deeper investigation to uncover their influence on research participation. While participants did not explicitly mention *exploitation or dissatisfaction with compensation*, cultural norms around discussing finances and stigma related to economic hardship may have contributed to hesitancy in voicing concerns. Therefore, perceptions of fairness in compensation should be interpreted with caution.

## Conclusion

We describe here the experiences, perceptions and ethical considerations related to deliberate malaria infection studies. Despite some debate on eligibility criteria, most participants agreed that the informed consent process was robust, and there was a strong sense of responsibility to contribute to the greater good. Overall the study reflects a complex interplay between personal motivations, ethical concerns, and the willingness to participate in controlled human infection model (CHIM) conducted elsewhere.

## Supplementary Information

Supplementary Material 1.

## Data Availability

Data underlying the paper may be requested from the Mahidol-Oxford Tropical Medicine Data Access Committee. (email: datasharing@tropmedres.ac)
