# Supplementary material for: Experiences, perceptions and ethical considerations of the malaria infection study in Thailand
Source: BMC Med Ethics. 2025 Jan 28;26:14. doi: 10.1186/s12910-024-01160-7 (PMC11773921; doi:10.1186/s12910-024-01160-7)
Supplement: Supplementary file 1 — Supplementary Material 1. [file 12910_2024_1160_MOESM1_ESM.pdf]

APPROVED
ETHICS COMMITTEE
FACULTY OF TROPICAL MEDICINE
MAHIDOL UNIVERSITY
Digitally signed by Faculty of Tropical Medicine, Mahidol University
DN: c=TH, st=Bangkok, l=Ratchathewi, o=Faculty of Tropical Medicine, Mahidol University, cn=Faculty of Tropical Medicine, Mahidol University, email=tmectropmed@mahidol.ac.th
Date: 2021.07.15 15:35:12 -07'00'

TOPIC GUIDE

Group 1

A. DURING RESIDENTIAL PERIOD

Familiarization with participants/Easing them into the discussion

Explore:

- How participants found out about the study, including sources of information that informs them about the study
- What information was given about the study, including expectations before arrival about how would happen (probe for screening/consent processes)
- What made them decide to come on the first day

Informed consent process

Explore:

- Whether participants feel they were given enough information during the info giving sessions
- Whether the information was easy to understand
- Whether they found any information challenging to understand
- What more information they would have wanted to get
- Whether they were allowed enough forums to ask questions?
- How they found the responses that were provided to the questions that were asked. How satisfying were the responses.
- Why some people were not included in the study? What they were told about the conditions under which someone could not be allowed to participate in the study?

Concepts of the 'deliberate infection'

Explore:

- Briefly what participants think the study entails
- What the study is aiming to find out?
- What they think about the idea of infecting people with an illness to observe them?
- Why they think it is important for such studies to be conducted?
- How they explained the study to family members and close network? Does the explanation relieve their concerns? How? (probe about any concerns that they had and how the participants address them)

- What people around you think about such a study? (probe about neighbours', friends', colleagues' or your social groups' views about challenge studies, potential supporters and those against, including reasons for such positions).

Motivating factors

Explore:

- What reasons/factors motivated participants to come to enroll in the study (probe for details for each reason mentioned, the reasons considered most important)
- What things they considered while making the decision to participate (time, procedures, compensation, medical care, anything else etc)
- Any specific things/issues that participants felt that if they were not addressed they would not have decided to participate in the study (probe for details of why the mentioned things/issues had to be addressed)
- Whether at any point during the study, they felt like withdrawing from the study. (if yes) Why? Thought about it but still decided to stay? Why?
- The things that can discourage them from participating in such study?

Residential period

Explore:

- Why the study requires to be stay at Hospital of Tropical Diseases?
- How prepared were the participants to stay in the hospital for such a long period? How did they plan to ensure that their stay did not become boring? (probe about: pre-departure plans and early in-resident coping strategies)
- Any benefits they experienced while staying at the Hospital of Tropical Diseases (probe: Which ones? Anything they liked)
- Any challenges they faced while staying at the Hospital of Tropical Diseases. Anything they disliked during your stay.
- What their feelings are about not being able to leave the Hospital of Tropical Diseases during the study
- Whether their family members raised any concerns regarding their stay at the Hospital of Tropical Diseases, including some of these issues/concerns raised.
- What the most difficult challenge has been while staying at the Hospital of Tropical Diseases, and how they have managed to address it.
- Whether if they are somethings that the research team might have done to make their stay in the Hospital of Tropical Diseases better.

(Probe for: Length of time; care by clinical staff; food options; entertainment; location; rooms)

APPROVED
ETHICS COMMITTEE
FACULTY OF TROPICAL MEDICINE
MAHIDOL UNIVERSITY

Experiences of participating in the study

Explore:

- What participants liked most about the study?
- What participants disliked most about the study?
- Which of the trial activities and procedures, were most challenging or difficult and Why *(Bleeding/blood volumes etc.)*
- Whether sometimes in course of the study they felt like completely withdrawing Why/why not?
- Whether they were any incidents that happened back home while they were in-residence that they felt like going back home (probe for: what happened; how the situation was eventually resolved?)
- About the things that motivated them to stay in the study to the end? (Probe for: financial compensation; availability of regular clinical care; support from other participants; good quality food etc.)
- Whether they Would recommend their friend/family member to participate in a similar study. Why/why not?
- What their overall experience has been since they joined the study. What expectations and fears they had at the begin. (probe for: expectations from clinical staff; other participants; managing the long-time in-residence; access to and visits from family/significant others)
- How they plan to use the financial compensation they will receive from participating in the study. (Probe for how they will ensure that such plans are realized; potential challenges in realising such plans; when and how the plans were arrived at?)

Permission to contact for follow-up interview

Inform participants that we plan to conduct interviews with some study participants after they leave the study (when they come back for follow). Ask, if it is okay to contact them later to ask if they will like to participate in the follow-up interview (For those who agree, get contact details and safely secure).

B. DURING FOLLOW SCHEDULE

Familiarisation with participants/Easing them into the discussion

Explore:

- How participants have been since leaving the study.
- What activities they have been engaging in since leaving the study.
- Whether they have had any engagement with MIST clinical team since leaving the study.

APPROVED
ETHICS COMMITTEE
FACULTY OF TROPICAL MEDICINE
MAHIDOL UNIVERSITY

Reflections about basic knowledge about the study

Explore:

- Whether they can still remember what the study was all about, including the main activities that participants had to undertake.
- What their reflections were about potential studies in general, especially the concept of deliberate infection in the context of clinical research.
- Whether the study has changed their views about clinical research (Probe: how and why).

Reflections about community perceptions about 'deliberate infection'

Explore:

- Whether they have shared their experiences about participating with some members within the community. What their views have been.
- What people in the community think about such a study following their interaction with community members since their return to the community (probe about wider community views about challenge studies, potential supporters and those against, including reasons for such positions).
- The common questions and issues that people are raising about those studies when they engage in discussions with them.
- How confident whether you are free from malaria in your body? Do you feel worry or concern whether you will be infecting other people?

Reflections about motivating factors for study participation

Explore:

- Their reflections about the factors that motivated them to participate and stay in the study. Looking back, will these still be the most important considerations for participating in such studies.
- Suppose a new challenge study was launched and they are invited to participate, will they still be motivated by the same factors.
- Why they considered those factors very important, and what people within the community think about the factors that participants mentioned.

Reflections about experiences of participating in the study

Explore:

APPROVED
ETHICS COMMITTEE
FACULTY OF TROPICAL MEDICINE
MAHIDOL UNIVERSITY

- Their reflections within the residential period during the study. What they liked most about their stay. What they disliked most about their stay. What could potentially be done to make the in-residence stay better.
- Their relationship with the study team and other participants while at the FTM hospital facility. Anything they learned during the study. Any possible way that knowledge that been of help since leaving the facility.
- Any concerns/worries they had before leaving the FTM hospital ward facility. What has become of the worries/concerns since their return home.

Reflections returning home and study compensation

Explore:

- How life has been since they returned home. How they have engaged in their regular activities.
- How their relationships with family, friends and other significant others have been since they returned home. How the relationships were when they returned and how have they evolved compared to when they were at the FTM hospital ward facility.
- Whether they have been able to undertake the activities they had planned to use their out of pocket financial compensation from the study for. How the experience has been and how the reaction from within their social networks have been?
- Looking back, were their expectations on how to use the money for realistic purpose? Whether any lessons have been learned in that process.

Future studies

Explore:

- Whether they would be willing to participate in a similar study (challenge) in the future.
- Whether they would participate, even if the level of compensation was less/none.
- Whether they would be willing to participate in a different type of study in the future. Why/why not?
  What are some of the things in future studies that can be done to make participants stay while in-residence better?

APPROVED
ETHICS COMMITTEE
FACULTY OF TROPICAL MEDICINE
MAHIDOL UNIVERSITY

Group 2

G. 2.1 People who express their interest in participating in but have not met the eligibility criteria for the MIST clinical studies

Concepts of the 'deliberate infection'

Explore:

- What participants think the study entails (probe for benefit, risk and long-term in patient stay)?
- What they know about deliberate malaria infection study of healthy human volunteers?
- What the study is aiming to find out?
- What they think about the idea of infecting people with an illness?
- Why they think it is important for such studies to be conducted?
- Why some people were not included in the study? What they were told about the conditions under which someone could not be allowed to participate in the study?

Motivating factors

Explore:

- What reasons/factors motivated participants to come to enrol in the study (probe for details for each reason mentioned, the reasons considered most important)
- What things they considered while making the decision to participate (time, procedures, compensation, medical care, anything else etc)
- Any specific things/issues that participants felt that if they were not addressed they would not have decided to participate in the study (probe for details of why the mentioned things/issues had to be addressed)
- The things that can discourage them from participating in such a study?

G. 2.2 Family and/or friends of Group 1 participants

Their roles and involvement in decision making

Explore:

- Who else they (Group 1 participants) involved in making the decision to join the study? Why them? Anyone else?
- What they (Group1 participants) told you regarding the study.
- What reasons/explanations they gave to their significant others for wanting to join the study and how they negotiated that decision
- Whether among those who felt they (Group 1) should not participate in the study, the reasons you provided. What they thought about the reasons given.

APPROVED
ETHICS COMMITTEE
FACULTY OF TROPICAL MEDICINE
MAHIDOL UNIVERSITY

- Factors that were considered before deciding to come join the study.
- Any concerns that were raised by family members regarding the study. Which ones?
- Whether they were some specific things they had to address before leaving home for the study.

<u>Relationship with clinical/Trial staff</u>

Explore:

- How they will describe their relationship with the clinical and research staff since they enrolled on the study (Probe for: respect, care and responsiveness while in-residence)
- Whether they have been able to raise issues/ask questions to the clinical staff. If they feel that they are available/accessible.
- If they felt that their questions and concerns were responded to adequately/in time. If they had any issues that were not responded to. Which ones? If they still require information regarding those specific questions and issues.
- How confident they are of the clinical care that they received while in-residence.

G. 2.3 General public: Focus Group Discussion

<u>Concepts of the 'deliberate infection'</u>

Explore:

- What participants think the study entails?
- What they know about deliberate malaria infection study of healthy human volunteers?
- What the study is aiming to find out?
- What they think about the idea of infecting people with an illness?
- Why they think it is important for such studies to be conducted?
- What people in the community think about such a study? (probe about wider community views about challenge studies, potential supporters and those against, including reasons for such positions).

APPROVED
ETHICS COMMITTEE
FACULTY OF TROPICAL MEDICINE
MAHIDOL UNIVERSITY

G. 2.4 Key Informants (knowledgeable and independent professional with recognised expertise): In-depth interview

Group 2.4: Key Informants including:

- malaria and infectious disease researchers/scientists
- public opinion influencers including public health workers, media representatives, lawyers, human rights activists, research ethics committee members
- staff involved in the MIST clinical study including staff from malaria laboratory, clinical laboratory and nurses

Intro/familiarization

- What is your role and responsibilities (what do they do in life; occupation)

Perceptions of the concept of 'deliberate infection' and MIST

- When did you hear about the term deliberate infection?
- In what capacity were you with CHIM when you know about this method the first time?
- What do you think about this method? Explore level of acceptance, any concern and how to overcome the concerns (e.g. safety, ethical issues)
- How will you explain about this method to people in the community?
- Do you think members of the public understand this method?
    - If yes, Probe:
        - what do you think they understand about the method?
    - If no, Probe:
        - how and what could you explain this method in a simple term?
        - how and what could we do to improve public understanding
- What do you think people in the community understand why this kind of method and MIST is important and why is it being undertaken?
- How challenge studies differ from other clinical studies such as clinical trial?
- Why challenge studies raise many social and ethical issues than other types of clinical studies?

Motivations for participation

- What do think are the motivations to participate in this study? Probe about the key issues/things that draws potential participants to participate in the study
- Whether if compensation was not being offered, people would still come to enroll for the study?

- What people in the communities are saying/think about the level of compensation for the study? Probe if it is perceived as an undue inducement that cannot be resisted, just okay or low.
- The extent to which challenge study participants focus so much on the proposed out of pocket payment than on the potential study risks. Probe for potential ways of bridging this gap in focus.

APPROVED
ETHICS COMMITTEE
FACULTY OF TROPICAL MEDICINE
MAHIDOL UNIVERSITY

Perception of long in-residential stay involved

- Potential issues that may arise from keeping participants away from their homes in an in-patient facility for a long time. Probe, about what individual, family and community perception of such practice.

Specific questions for ethics committee members

- Why challenge studies generally raise many ethical issues. Probe on specific ethical issues that have been raised within ongoing and planned challenge studies in Thailand
- What the prevailing guiding frameworks for conducting challenge studies globally are. To what extent the existing guidelines/frameworks meet the challenges faced by various challenge study stakeholders in Thailand (probe for what works and what does not work).
- Considering that many research ethics committees and members may not be familiar with challenge study, what are the perspectives about the installation of nationally 'flagged' research ethics committees with capacity to review challenge studies. Probe the possible advantages and disadvantages of such a review system for challenge studies.
- The appropriateness, accessibility and gaps in current ethical guidelines (local and international). Probe whether there is the need to explore developing local challenge study guidelines, including the process for the development of such guidelines.
- The unique ethical and regulatory challenges or dilemmas that they have experienced with challenge studies (Probe how they work towards addressing the challenges/dilemmas).

Specific questions for MIST study team

- Research team's experiences with research ethics committee(s) responsible for reviewing and approving challenge studies been? (probe how the interactions and responses have been over time). Whether there is a need to have an informal discussion with some members of the committee before submitting the protocol. How they have found research ethics committees understanding and appreciation of challenge studies.
- How practicing clinicians reconcile the fact that challenge studies involve deliberately 'harming' someone with an infectious agent (Arguably incompatible with the Hippocratic oath of 'do no harm'). How study clinicians grapple with such issues. How that could potentially impact on clinician-patient relationship in the future.

Specific questions for recruitment team (FTM-CTU Team)

Explore: Ensuring fair participants recruitment

- Could you please share with us your experience of recruiting and caring participants (Probe: any difficulties, worries and any memorable incidents)?
- How did you prepare yourself in working in this project?
- Considering that many people perceive the out of pocket expenses provided to challenge study participants as relatively high compared to some local jobs, what mechanisms are in place to ensure that recruitment of potential participants into challenge studies do not unfairly 'attract' a particular group of persons (e.g. Unemployed or self-employed) or 'creates' a group of 'professional research participants'? (Probe for details of the various strategies mentioned).

APPROVED
ETHICS COMMITTEE
FACULTY OF TROPICAL MEDICINE
MAHIDOL UNIVERSITY

- Whether they have experienced situations where some participants perceive the recruitment process into a challenge study as unfair (Probe how such concerns have been resolved)
- With the research team how potential participants react when they are informed, they are unable to participate in the challenge study for different reasons (Probe how they handle such situations are handled).

APPROVED
ETHICS COMMITTEE
FACULTY OF TROPICAL MEDICINE
MAHIDOL UNIVERSITY
